# IS-Linked Movement of a Restriction-Modification System

**DOI:** 10.1371/journal.pone.0016554

**Published:** 2011-01-31

**Authors:** Noriko Takahashi, Seishi Ohashi, Marat R. Sadykov, Yoko Mizutani-Ui, Ichizo Kobayashi

**Affiliations:** 1 Laboratory of Social Genome Science, Department of Medical Genome Sciences, Graduate School of Frontier Science, University of Tokyo, Minato-ku, Tokyo, Japan; 2 Institute of Medical Science, University of Tokyo, Minato-ku, Tokyo, Japan; 3 Graduate Program in Biophysics and Biochemistry, Graduate School of Science, University of Tokyo, Minato-ku, Tokyo, Japan; University of Florida, United States of America

## Abstract

Potential mobility of restriction-modification systems has been suggested by evolutionary/bioinformatic analysis of prokaryotic genomes. Here we demonstrate in vivo movement of a restriction-modification system within a genome under a laboratory condition. After blocking replication of a temperature-sensitive plasmid carrying a PaeR7I restriction-modification system in *Escherichia coli* cells, the plasmid was found integrated into the chromosome of the surviving cells. Sequence analysis revealed that, in the majority of products, the restriction-modification system was linked to chromosomal insertion sequences (ISs). Three types of products were: (I) apparent co-integration of the plasmid and the chromosome at a chromosomal IS1 or IS5 copy (24/28 analyzed); (II) *de novo* insertion of IS1 with the entire plasmid except for a 1–3 bp terminal deletion (2/28); and (III) reciprocal crossing-over between the plasmid and the chromosome involving 1–3 bp of sequence identity (2/28). An R-negative mutation apparently decreased the efficiency of successful integration by two orders of magnitude. Reconstruction experiments demonstrated that the restriction-dependence was mainly due to selection against cells without proper integration: their growth was inhibited by the restriction enzyme action. These results demonstrate collaboration of a mobile element and a restriction-modification system for successful joint migration. This collaboration may have promoted the spread and, therefore, the long-term persistence of these complexes and restriction-modification systems in a wide range of prokaryotes.

## Introduction

**Figure 1:**
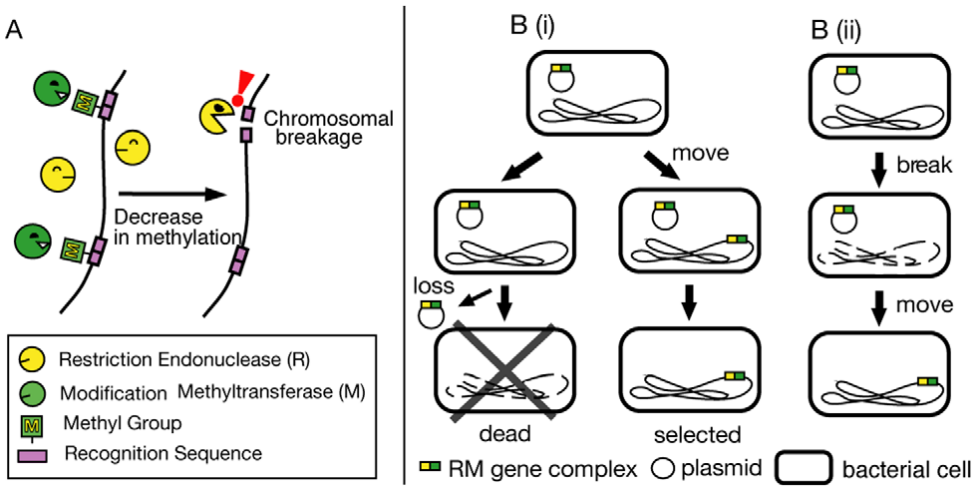
Probable actions of restriction-modification (RM) systems. **A.** Post-segregational killing. Loss or disturbance of an RM system leads to chromosomal breakage by the R enzyme and, unless repaired, to cell death. **B.** Two possible roles of R in the successful movement. **(i)** Selection. The RM system on a plasmid moves to a new locus in the genome. The cells with the unrearranged genome are killed by RM attack after the plasmid loss, while the cells carrying the chromosome into which RM is incorporated would survive. **(ii)** Movement. RM-mediated DNA breakage promotes movement.

A restriction (R) endonuclease recognizes a specific DNA sequence and introduces a break on the DNA. A cognate modification (M) enzyme can methylate the same sequence, and thus protect the DNA from cleavage. The tight association of an R enzyme gene with a cognate M enzyme gene has been termed as an RM system. R enzymes cleave foreign DNA if its recognition sequences are not properly modified. RM systems are thought to be maintained by prokaryotes as defensive tools against invasion by foreign DNA. However, there is evidence that certain RM systems may behave as a selfish mobile genetic element and attack host bacteria [1], [2]. Several type II RM gene complexes resist replacement by a competitor genetic element [3], [4], [5] by killing cells that have lost the RM gene complex, through cleavage of the chromosome by the remaining R enzyme molecules, a process known as post-segregational host killing (Figure 1A) [3], [6], [7], [8], [9]. Evolutionary game theory analysis demonstrated that such selfish genes can spread in the presence of spatial structure [8].

Mobility and horizontal transfer seem to be general features of the selfish genetic elements, and to be important for their long-term persistence at a broader level [10], [11], [12]. Various lines of evidence support the mobility of RM genes over different time scales [1], [2], [13], [14]. Their extensive horizontal transfer between distant groups of prokaryotes is suggested by phylogenetic analysis and bioinformatic analysis of GC content, codon usage, and word frequency. Comparison of two closely related bacterial genome sequences suggested that all types of RM systems can insert themselves into a genome, generating long and variable target duplications [13], [15] and that they are linked to an inversion or a translocation [16], [17]. Many RM genes occur on mobile genetic elements, such as plasmids, prophages, transposons, conjugative transposons, integrons and operons [1], [2], [13], [18], [19]. Several RM gene complexes are flanked by insertion sequence (IS) repeats and might move as a composite transposon [13], [20], [21]. Maintenance of these mobile units may be stabilized by the presence of the RM system in a similar way as was found for superintegrons [22]. Similar wide-spread occurrence was observed for toxin-antitoxin loci [23].

The mobility and rearrangements could be related to the R activity in two possible ways. The R-mediated breakage might cause the DNA rearrangement (Figure 1B (ii)) [24], [25]. Alternatively, genomes in which particular rearrangements have not taken place might be destroyed by R activity (Figure 1B (i)). This implies selection of the rearranged genomes by the RM system.

In this work, we detected *in vivo* movement of an RM system within bacterial cells under a laboratory condition. The movement was associated with an IS copy in most cases, and was apparently dependent on R activity. Our reconstruction experiments demonstrated that the R-dependence was mainly due to the selection.

## Results

### Detection of movement of an RM system

**Figure 2:**
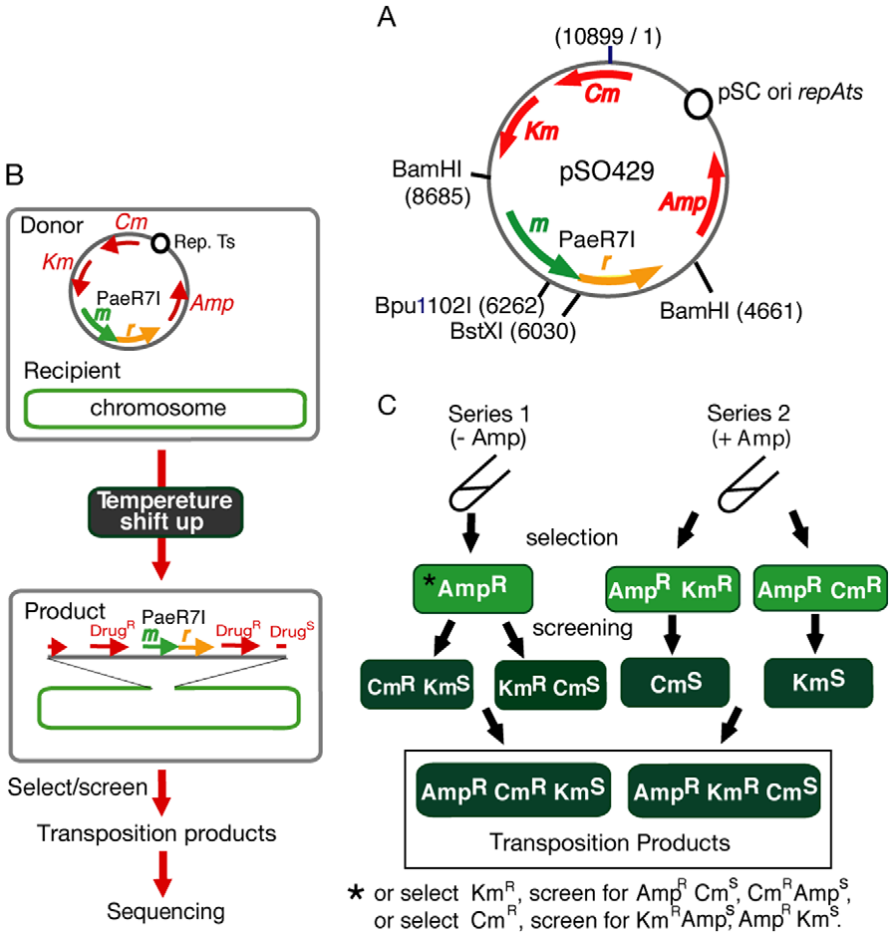
Experimental design for detection of RM gene complex movement. **A.** The donor plasmid pSO429, which relies on temperature-sensitive (pSC101-derived) replication machinery, carries the PaeR7I RM gene complex and three drug-resistance genes (Amp^r^, Km^r^ and Cm^r^). **B.** The plasmid was introduced into *E. coli*. MG1655 strain. The shift to the temperature non-permissive for replication induce cell death due to post-segregational killing, and would allow growth of the cells carrying the plasmid harboring PaeR7I RM genes.**C.** In the absence of ampicillin (series 1) or in its presence (series 2), cells retaining at least one drug-resistance gene (Amp^r^ or Km^r^) and missing one (series 1), or cells retaining two drug-resistance genes (Amp^r^ and Kan^r^, or Amp^r^ and Cm^r^) and missing one (series 2) were selected and screened at 42°C on solid medium. The double drug-resistant clones (three types in series 1 and two types in series 2) obtained from the above process were subjected to sequencing to determine the integration point on the chromosome.

To detect movement of an RM system, we designed an assay based on post-segregational killing caused by PaeR7I RM system (Figure 2) [3], [26],[27]. This type II RM system recognizes a palindromic sequence 5′CTCGAG, where the restriction endonuclease cleaves between the first and second base and the methyltransferase modifies the fifth base to 6-methyladenine (http://rebase.neb.com). The donor plasmid (pSO429) (Figure 2A, Table S2) carries the PaeR7I RM gene complex and three drug-resistance genes for ampicillin (Amp^r^), kanamycin (Km^r^), and chloramphenicol (Cm^r^), respectively. Its replication is controlled by a thermo-sensitive replication initiator [28]. The R-negative (R^−^) control plasmid (pSO431) carries a mutation in the restriction gene (Table S2). The plasmid was maintained in *Escherichia coli* strain MG1655, whose complete genome sequence has been published [29], at a replication-permissive temperature (30°C). We tried to detect movement of the RM system together with one or two of these drug-resistance genes to the chromosome. To minimize homologous recombination between the donor plasmid and the recipient chromosome, the donor plasmid was designed [30] to lack DNA sequence identity longer than 14 bp with the chromosome (see Table S2). To access this goal, we performed two series of experiments.

#### Experiment series 1

The procedure is essentially the same as that used to analyze post-segregational killing by RM systems [3], [7].

The cells were grown in a liquid medium with shaking at a low temperature that allows replication of the plasmid (30°C) with the antibiotics selective for the three drug-resistance genes. Then the selective antibiotics were removed and the temperature was shifted to 42°C, a higher temperature that inhibits the replication of the plasmid. Total viable cells were detected as colony forming units on agar lacking any antibiotics at 30°C. Viable cells carrying the plasmid were detected as colony forming units on agar containing the selective antibiotics at 30°C (as indicated in Figure 2C). In the latter case, the plasmid may be extra-chromosomal or integrated.

**Figure 3:**
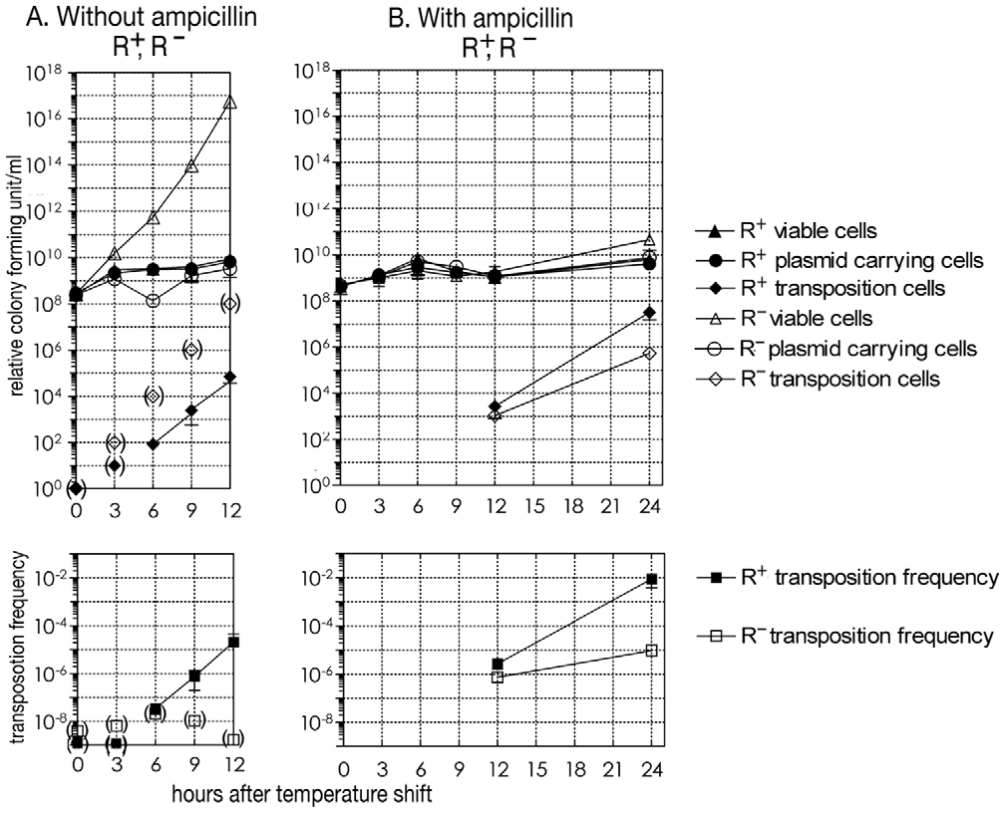
Detection of PaeR7I RM gene complex movement after replication block. **A.** (Series 1) *E. coli* cells carrying pSO429 (pSC101ts, PaeR7I R^+^M^+^, Amp^r^, Km^r^ and Cm^r^) or pSO431 (the R^−^ version) were isolated and grown to log phase at 30°C with the three selective antibiotics. Cells were then centrifuged and re-suspended in LB broth at 42°C without antibiotics (hour 0). After incubation with aeration at 42°C, an aliquot was spread on agar containing antibiotics and incubated overnight at 42°C in order to select and screen for transposition clones as described in Figure 2. Viable cells were calculated from the number of colony formers on LB agar without selective antibiotics after incubation at 30°C. Plasmid-carrying cells were calculated from the number of colony formers on LB agar with selective antibiotics after incubation at 30°C. Transposition frequencies were calculated as (number of double drug-resistant cells per ml)/(number of viable cells per ml). Average values of six independent tubes were plotted. **B.** (Series 2) *E. coli* cells carrying pSO429 (R^+^) or pSO431 (R^−^) were grown and prepared for the temperature shift as in A. At hour 0, cells were centrifuged and re-suspended in LB broth containing a low dose (25 µg/ml) of Amp at 42°C, and incubated with aeration at 42°C. Aliquots were subjected to selection and screening as shown in Figure 2C and the transposition frequency, as well as the number of viable cells, plasmid-carrying cells were calculated as in A. The averages of 5 (R^+^) or 4 (R^−^) measurements were plotted. The upper graphs show relative colony forming unit and the lower graphs show calculated transposition frequency.

As shown in Figure 3A, in the culture of cells carrying the restriction-negative (R^−^) control plasmid, the temperature shift stopped increase in the number of plasmid-carrying cells after a lag as expected. However, the viable cell counts continued increasing. During the course of the experiment, the culture was appropriately diluted to avoid saturation, so that shown in figure 3A is the relative cell concentration in the absence of the dilution process.

In the R-positive (R^+^) case, the temperature shift stopped the increase in viable cell count as well as the number of plasmid-carrying viable cells, as reported (Figure 3A) [3], [7]. This is due to the post-segregational killing process by the RM system described above.

Under this condition, we looked for products of movement of the RM system together with one or two of the drug-resistance genes from the plasmid to the chromosome, as illustrated in Figure 2B. We first selected clones (colonies) that were resistant to the higher temperature and carrying one of the adjacent antibiotic resistant genes (Amp^r^ or Km^r^), and then screened them for loss of resistance to one of the other two antibiotics (Figure 2C). These selected and screened clones were tentatively defined as transposition clone candidates (Table S1). As shown in Figure 3A (filled square), these transposition cells were detectable 6 hrs after the temperature shift and increased in R^+^ culture.

We further asked whether their appearance was dependent on the restriction function or not. However, in the R^−^ culture, we could not sensitively measure these transposition products because of the growth of the cells that had lost the plasmid (Figure 3A, open triangle in the upper panel and open square in the lower panel). In order to examine dependence on the restriction function, we performed experiment series 2.

#### Experiment series 2

Figure 3A (experiment series 1) shows that the stop of the viable cell count because of the post-segregational killing in the R^+^ culture allowed us to detect the transposition clones. In order to eliminate cells that no longer carried the plasmid but continued to grow in the R^−^ culture, a low dose of Amp (25 µg/ml) was added at the start of the temperature shift (hour 0) in experiment series 2 (Figure 3B), and then the culture was appropriately diluted as in series 1. The resulting viable cell count curve was indistinguishable between R^+^ and R^−^ (Figure 3B). This system enabled us to measure and compare the number of transposition products by the selection/screening procedure in the two genetic backgrounds. Addition of Amp restricted the detectable transposition clones to Amp^r^ Cm^r^ Km^s^ and Amp^r^ Cm^s^ Km^r^. Thus, the colonies that were resistant both to the higher temperature and two of the antibiotics (Amp and Cm, or Amp and Km) were screened for loss of resistance to the remaining antibiotic (Km or Cm) (Figure 2C, experiment series 2).

In this way, we were able to detect transposition products for the R^−^ cultures (Figure 3B, upper panel, open square). The number of these transposition clones and the transposition frequency (number of double resistant cell per ml at 42°C/number of viable cell per ml at 30°C) (Figure 3B, lower panel) were comparable between R^+^ and R^−^ cultures at hour 12. However, at hour 24, the frequency in the R^+^ culture became at least two orders of magnitude higher than that in the R^−^ culture (Figure 3B, lower panel). In other words, there appeared strong dependence on restriction function, to which we will come back after examination of structure of these products.

### Product structure at the sequence level

All examined RM transposition candidate clones lost one drug resistance gene, but retained the other two, resulting in Amp^s^ Km^r^ Cm^r^, Km^s^ Cm^r^ Amp^r^ or Cm^s^ Amp^r^ Km^r^ phenotype (experiment series 1), or in Cm^r^ Amp^r^ or Cm^s^ Amp^r^ Km^r^ phenotype (experiment series 2). In the next steps, we analyzed only one clone for the same drug-resistance pattern from each culture tube to assure independence of the clones.

In experiment series 1, the products from R^+^ cultures were examined for restriction modification phenotype in a phage restriction assay. All examined (38/38) turned out to be R^+^, and all examined (13/13) turned out to be modification-positive. In experiment series 2 experiments, all the examined products from R^+^ cultures (7/7) remained R^+^ and all from R^−^ cultures (12/12) remained R^−^.

The starting plasmid carries a single BstXI site and a single Bpu1102I site within PaeR7I RM system (Figure 2A). If a single copy of the plasmid has integrated into various locations along the chromosome, Southern hybridization after cleavage with one of these enzymes, with the BamHI/BamHI PaeR7 RM fragment (Figure 3B) as a probe, would give two bands of various lengths. This prediction was verified for all (20/20) non-sister R^+^ transposition clones from experiment series 1 (Bpu1102I and BstXI, data not shown), 6/6 R^+^ clones from experiment series 2 (Bpu1102I, BstXI) and 12/12 R^−^ clones from experiment series 2 (Bpu1102I) (Figure S1). Because the BstXI site was inactivated by the R^−^ mutation, a single band was seen with BstXI for all (12/12) of the R^−^ clones (data not shown). Extra-chromosomal plasmids were not detected in any of the examined clones (10/10 for R^+^ and 10/10 for R^−^ in experiment series 2) by Southern analysis using the PaeR7 RM probe (data not shown).

**Figure 4:**
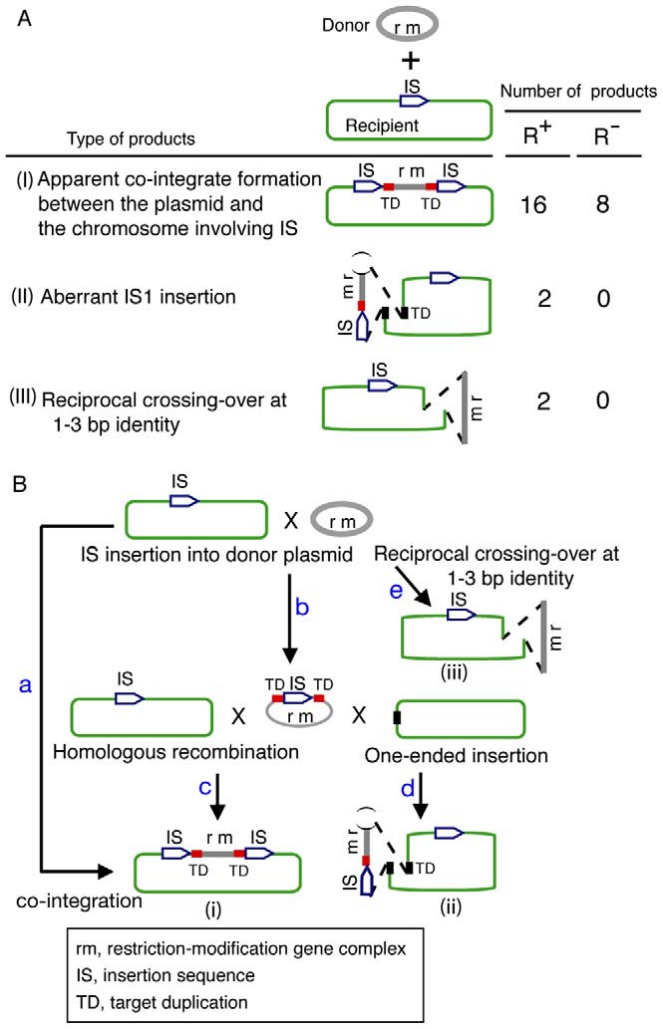
Product structure. **A**. Three types of transposition products. (I) *Apparent co-integrate*. Apparent co-integration of the plasmid and the chromosome at IS1 or IS5 already present on the chromosome. IS1 integration was associated with target duplication (TD in red) of 8–10 bp at various donor (plasmid) sites. IS5 generated a 4-bp target duplication at various donor sites (Figures S2and S3). (II) *Aberrant IS insertion*. *De novo* insertion of IS1 with the entire plasmid, except for a 1- or 3-bp terminal deletion (black parentheses). The chromosomal locus had a 9-bp target duplication. The right end of the plasmid insert was similar to the end of IS1 in one product (Figure S4). (III) *Reciprocal recombination*. Reciprocal crossing-over between the plasmid and the chromosome involving 1- or 3-bp region of sequence identity (Figure S5). **B.** Hypothetical routes to the products. (a) One-step co-integrate formation between the chromosome and the plasmid at a chromosomal IS. (b) Chromosomal IS insertion into the RM plasmid. (c) Homologous recombination between the plasmid IS and a homologous chromosomal IS leading to the apparent co-integrate. (d) Plasmid IS experiences one-ended transposition into a new chromosomal target site with target duplication. (e) Plasmid and chromosome undergo reciprocal illegitimate recombination at the 1–3 bp identity. **Symbols:** green, chromosome; white arrow, IS; gray, plasmid; red, plasmid target duplication; black, chromosomal target duplication.

We then amplified DNA flanking both ends of the integrated plasmid sequence by inverse PCR [31], for each of the 28 transposition products (Table S1). The amplified DNA was used for direct sequencing (see Materials and methods). The products were classified into three types at the sequence level (Figure 4, Figure S2).

Type I, the majority (16/20 analyzed) (Figure 4A (I), Figure S2, S3 left) of the sequenced transposition clones, showed apparent co-integration of the plasmid and the chromosome at an IS1 or IS5 already present on the chromosome. IS1 co-integration was associated with about 8–10 bp target duplication, as reported [32], [33] at various donor sites. Of seven IS1 copies in the MG1655 chromosome, only two were involved, with 10 clones at an IS1 copy at position 19796 and 9 from an IS1 at position 1996527. IS5 generated 4-bp target duplication, as reported [34], at various sites. All (8/8) examined products from experiment series 2 including the transposition products from R^-^ control were of this type (Figure S3 right). Formation of this type was readily explained by co-integarate formation between the plasmid and the chromosome at the chromosomal IS copy (Figure 4B, a), although other possibilities will be discussed later.

Type II products showed aberrant IS insertion (2/28) (Figure 4A (II), Figure S4). This type was characterized by *de novo* insertion of IS1 with the entire plasmid, except for a very short (1 or 3 bp) terminal plasmid deletion. The chromosomal locus had a 9 bp target duplication. This type of products can be explained by transposition of a chromosomal IS into the plasmid (Figure 4B, b) followed by aberrant, ‘one-ended’ insertion into a novel site in the genome with target duplication (Figure 4B, d) as discussed later.

Type III products (2/28) appeared to have been generated by reciprocal illegitimate recombination events (Figure 4A (III), Figure S5). Reciprocal crossing-over occurred between the plasmid and the chromosome at a region of very short (1 or 3 bp) sequence identity. Both of the events involved 5′ CCATT in the donor plasmid, for unknown reasons. The possible origin of these three types will be discussed later.

### Reconstruction of restriction-dependent selection against cells without proper transposition

Two possible hypotheses may explain the restriction dependence observed at hour 24 after the temperature shift (Figure 1B). One is contribution of restriction enzyme action to generation of transposition products in some way (Figure 1B (ii)). The other is relative growth advantage of the preformed transposition product cells over the other cells that is greater in the R^+^ culture than in the R^−^ culture; in other words, selection of the transposition product cells (Figure 1B (i)). A likely process underlying the selection is restriction-enzyme-mediated killing or growth inhibition of cells without transposition.

There is no significant difference in the frequency of the transposition products at hour 12 after the temperature shift (Figure 1B). If the selection is operating, the transposition lines already present at hour 12 would increase in frequency at hour 24.

To examine this possibility, we carried out the following reconstruction experiments. We picked and marked one R^+^ Amp^r^ Cm^r^ transposition product clone (and one R^−^ Amp^r^ Cm^r^ transposition product clone) with a tetracycline-resistance gene (Tet^r^) (Table S1). We added approximately 250 cells of the R^+^ (or R^−^) Tet^r^ labeled transposition products (grown to log phase) to 5 ml of temperature-shifted culture (R^+^ or R^−^) at hour 12, and counted the number of Amp^r^ Cm^r^ transposition products and Tet^r^ colony formers (preformed transposition products) at hour 24.

**Figure 5:**
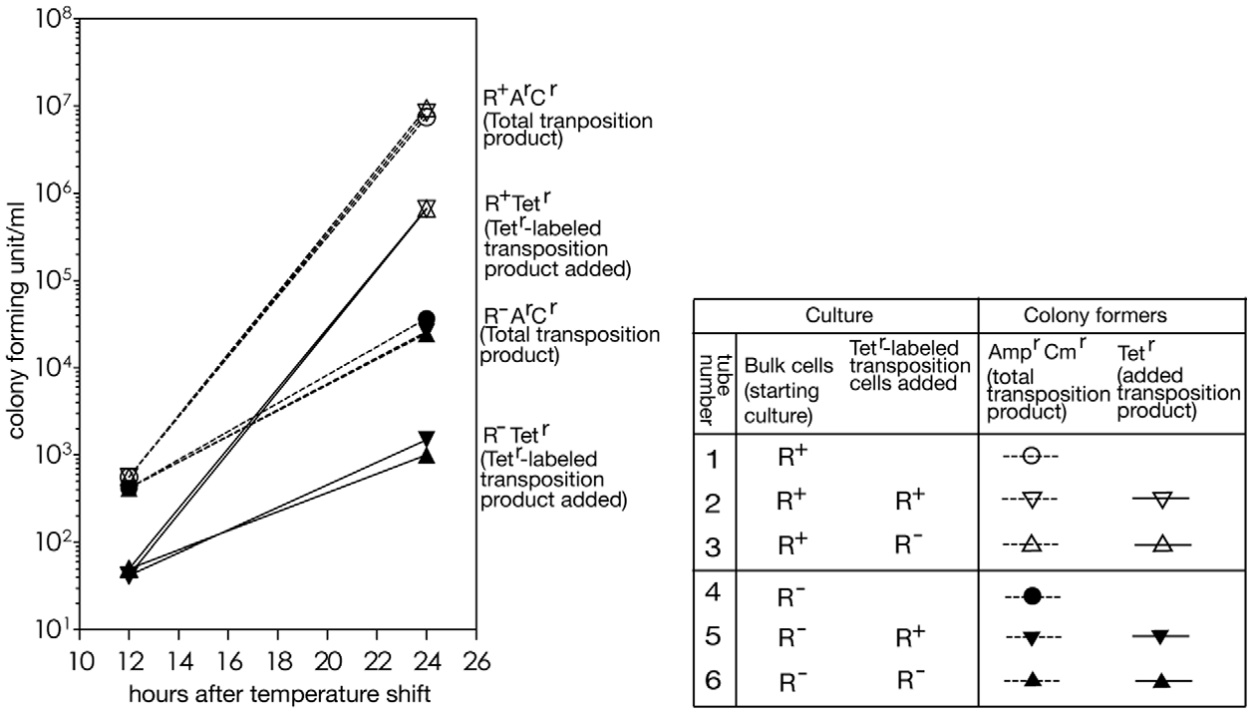
Reconstruction experiment demonstrating selective advantage of transposition products in an R-positive culture. One transposition clone from an R-positive (R^+^) culture and one from an R-negative (R^−^) culture were labeled with the Tet^r^ gene (materials and methods). Temperature-shift experiment was performed as described in Figure 3B, in the presence of Amp, with Tet^s^ cells carrying an R^+^ (Tube 1) or R^−^ (Tube 4) ts plasmid. At hour 12, about 50/ml of Tet^r^-labeled transposition cells was added to portions from tube 1 to make tube 2 (Tet^r^-labeled R^+^ clone added) and tube 3 (Tet^r^-labeled R^−^ clone added), and to portions from tube 4 to make tube 5 (Tet^r^-labeled R^+^ clone added) and tube 6 (Tet^r^-labeled R^−^ clone added). All 6 tubes were aerated at 42°C until hour 24. Bulk transposition cells (dotted lines) were measured as colonies that grew on LB agar with Amp (with Oxa) and Cm at 42°C, but not on Kan agar, as for Figure 3B. Tet^r^-labeled transposition cells (solid lines) were measured as colony formers on LB agar supplemented with Tet at 42°C. The culture was diluted twice to avoid entrance to saturation. The relative concentrations of colony formers in the absence of the dilution were calculated and plotted. The average values from two independent tubes were plotted.

The results of the reconstruction experiments are shown in Figure 5. Our observations and their interpretations are summarized as follows.

- The increase, from hour 12 to hour 24, of the frequency of the Tet^r^ labeled transposition products was more in the R^+^ culture than in the R^−^ culture (Tubes 2 and 6). This suggests that there is restriction-dependent selection for the transposition products (against the R^+^ parental clones).
- The increase was determined by the restriction genotype of the bulk culture but not by the restriction genotype of the transposition products. The R^−^ transposition products and the R^+^ transposition products showed the same higher rate of increase in the frequency when added to the R^+^ culture (Tubes 2 and 3). The R^−^ and R^+^ transposition products showed the same lower rate of increase in the frequency when added to the R^+^ culture (Tubes 6 and 5). This indicates that the selection is due to the slow growth of the majority of the cells in the R^+^ culture.
- The frequency of the bulk, unlabeled Amp^r^ Cm^r^ transposition products in the R^+^ culture increased between hour 12 and hour 24 (Figure 5, Tubes 1, 2 and 3) as in the previous experiment (Figure 3B). The increase was much lower in the R^−^ culture (Figure 5, Tubes 4, 5 and 6). These indicate R-dependence. The increases in the transposition products closely paralleled the increase in the Tet^r^-labeled transposition products in the R^+^ cultures and in the R^−^ cultures. Therefore, the R-dependent increase can be explained by selection, that is growth advantage of the transposition products in the R^+^ cultures.

The above results strongly suggest that the difference in transposition frequency between the R^+^ culture and the R^−^ culture at hour 24 (Figure 3B) is mainly ascribed to the difference, in the further growth of the transposition products already present at hour 12, between the two cultures. The R-dependent increase of the transposition product from hour 12 to hour 24 (Figure 3B) was likely due to selection against the cells without transposition through restriction enzyme action on them (Figure 1B (i)). There is no evidence for *de novo* formation of the transposition products during this period.

The likely process underlying the selection is R-mediated inhibition of growth of the cells without RM transposition through the cleavage of chromosome without sufficient protection of restriction sites due to the absence of methylase as illustrated in Figure 1B (i).

## Discussion

Our results demonstrate intracellular movement of an RM gene complex, for the first time under laboratory conditions (Figure 3). The majority of the products showed IS-associated co-integrate of the plasmid and the chromosome (Figure 4A). Our reconstruction experiments (Figure 5) indicated that the R-dependency be mainly ascribed to selection against cells with the parental genotype in the R^+^ culture, likely through attack on their genome (Figures 1A and B (ii)).

### Role of restriction activity

The R-dependence through selection is explained by the post-segregational killing process (Figure 1B (i)) [3], [6], [7], [26]. The replication block of the RM plasmid by the temperature shift decreased its copy number, leading to a decrease in the number of RM molecules, which in turn caused R-mediated breakage of insufficiently methylated genomic sites (Figure 1A, B), leading to the cell death process.

The R-mediated death or growth inhibition here may be a complex process. Damage induces the SOS response [7]. Homologous recombination repairs the break [7], and initiates DnaA-independent DNA replication [35], which exposes more unmethylated R targets. SOS induction inhibits cell division and generates multi-nucleated filamentous cells [7], [26], which we confirmed under our conditions (N.T. and I.K., unpublished). In these filaments, the ratio of the plasmid copy number to the chromosome copy number is smaller than unity, and thus the concentration of the modification enzyme would be relatively low. Some cells will lose the last copy of the plasmid and suffer further chromosomal restriction breakage and consequent cell death. Instability of the M enzyme may accelerate these processes [36], [37]. We observed broken chromosomes even at 24 hours after the temperature shift (Figure S6). A recent analysis revealed dynamic transcriptome changes after loss of the PaeR7I RM gene complex, culminating in cell lysis and protein release [26]. Genome breakage likely triggers programmed death pathway intrinsic to bacterial cells [26].

Cells into which the RM genes integrated the chromosome would retain genome methylation, and therefore would not experience chromosome damage and cell death. Its healthy state was indicated by our observation that added Tet^r^-labeled R^+^ integration product grew as fast as added Tet^r^-labeled R^−^ integration product in the R^+^ culture (Figure 5, Tubes 2 and 3) and by themselves (N.T. and I.K., unpublished).

In the reconstruction experiment in Figure 5, we did not obtain evidence for contribution of restriction to *de novo* transposition, because of the dominant selection effect of restriction. However, we cannot exclude the possibility that R enzyme action also contributed to generation of the integration products as well. Type II R-enzyme action stimulates homologous recombination in various ways [24], [25]. Addition of mitomycin C, a DNA-damaging agent known to cause chromosomal breakage and induce the SOS response, increased the number of transposition products by four orders of magnitude in the R-negative culture and one order of magnitude in the R-positive culture (Table S4). There could be several possible reasons for the inability of the restriction enzyme to significantly stimulate the transposition. These include the paucity of their recognition sites along the chromosome, repair of the breakage by recombination, and absence of the plasmid molecule from the cell during the post-segregational killing process.

### Formation of the products

Sequence-level structure suggests molecular mechanisms for formation of the three types of integration products. Type I, apparent co-integration, was the most frequently encountered product type (Figure 4A (i)). They could be products of classical transposase-mediated co-integration (Figure 4B a). However, while IS1 forms co-integrates and simple inserts in comparable numbers, IS5 forms only simple inserts [38]. An alternative route is illustrated in Figure 4B, in which the chromosomal IS becomes inserted into the plasmid (Figure 4B b). Then, homologous recombination (reciprocal crossing-over) between the plasmid IS and a chromosomal IS of the same family generates the co-integrate form (Figure 4B c). Indeed, crossing-over between ectopic chromosomal ISs occurs under a similar condition [4]. The biased use of IS copies on the chromosome (Table S1) may reflect their differential activity in co-integrate formation in the first route, or in insertion formation in the second route [39], and/or the distribution of hot spots for homologous recombination in the chromosome in the second route. The latter, two-step route can be demonstrated when the two IS copies in the second step have different sequences. However, we were unable to find such a case in our sequence analysis.

The product form of an RM gene complex flanked by direct IS repeats is interesting in two respects. First, this complex may behave as a composite transposon and efficiently move to new loci. An RM gene complex flanked by inverted IS repeats was described [21], and recent studies revealed that several RM gene complexes are flanked by direct IS repeats [13], [20]. Second, this form is expected to be able to increase its copy number by tandem amplification through apparent unequal crossing-over between the direct repeats, as demonstrated previously [5]. This form is thus similar to those generated by insertion by long and variable target duplication [13], [15].

It is noteworthy that when we used the IS-less strain ((MDS42) [40], purchased from Scarab Genomics) as a host, essentially no double-resistant colony was detected both in R^+^ case and R^−^ case.

Type II was *de novo* IS-plasmid insertion. In this second product type, *de novo* insertion of a chromosomal IS copy with a terminally deleted plasmid molecule (Figure 4A (II), Figure S4) may be explained by one-ended insertion (Figure 4B d) [41]–[42] from a plasmid molecule carrying an IS insertion, which was hypothesized in the above two-step route for the co-integrate type. Indeed, the right end of the plasmid insert shows similarity (14/23 bp identity) to the right end of IS1 in one of the products (Figure S4).

Type III, IS-free products (Figure 4A (III)) could be explained by reciprocal recombination involving 1 or 3 bp of sequence identity (Figure S5). We do not know whether these arose through a break-join or a replication-error mechanism. We also do not know why both cases involved 5′ CCATT, which is quite different from the PaeR7I recognition sequence, 5′ CTCGAG. We cannot exclude the possibility that these products are also related to the action of ISs, although the sequences involved appear unrelated to IS1 or IS5.

We did not detect any relationship between the distribution of the recombination sites in the above types of products, PaeR7I sites, or chi sites (data not shown).

### Collaboration of post-segregational killing by RM systems and mobile elements

Earlier we showed that type II RM systems examined use R enzyme activity to promote short-term maintenance by killing cells that have failed to maintain them [3], [8]. In this work we demonstrate that this activity allows selection of a rearranged genome in which the elements moved to a new locus and expressed properly. The intra-genome movement was mediated by a mobile IS element in most cases. Acceleration of intra-genome and, by extrapolation, inter-genome mobility may be possible through similar collaboration or symbiosis between RM systems and other types of mobile elements. Mobile elements propagate in a new genome environment more efficiently by destruction of their previous form, a potential competitor. Therefore, our results extend the concept of post-segregational killing to gene transfer. The potential of host cell attack and symbiosis with mobile elements likely promoted the spread, and therefore, the long-term persistence of RM complexes in a wide range of bacteria [1], [2], [13]. The same could be true for other post-segregational (toxin-antitoxin) systems [23].

## Materials and Methods

### Bacterial strains and plasmids (see also Table S2)

MG1655, an *Eschechia coli* strain whose genome has been entirely sequenced [17], is a gift from Dr. Don Biek. Plasmid pSO421 was constructed from pHSG415 [19] by removing a 159-bp long region homologous to the *E.coli* chromosome. The donor plasmid pSO429 (Figure 2A) was constructed by inserting a 4.0 kb BamHI fragment containing the PaeR7I R ^+^ M^+^ gene complex from pTN5 [2] into a BamHI site of pSO421. pSO431 is its PaeR7I R^−^ M^+^ version, constructed by inserting a KpnI linker (5′gggtaccc) into the filled-in BstXI site of the PaeR7I R gene. *E. coli* cells were grown in LB (Luria-Bertani) broth [4] supplemented, if necessary, with antibiotics at the following concentrations: Amp at 50 µg/ml (unless otherwise stated); Oxa at 150 µg/ml; Cm at 15 µg/ml; Km at 50 µg/ml; Tet at 12.5 µg/ml.

### Temperature shift experiment

Single colony was isolated from *E. coli* MG1655 carrying pSO429 and MG1655 carrying pSO431 by streaking on LB agar containing Amp, Oxa, Cm, and Km and incubating overnight at 30°C. Colonies were inoculated into L broth containing Amp, Oxa, Cm, and Km and cultured overnight at 30°C. Next day, overnight cultures were diluted to 5×10^7^ cells/ml in L broth containing Amp, Oxa, Cm, and Km, and grown to log phase (1–5×10^8^ cells/ml) at 30°C. These cultures were then spun down for 10 min at 3.5 krpm at 4°C, and the obtained cell pellets were immediately resuspended in 5 ml of LB broth without antibiotics addition in experiment series 1, and with Amp (25 µg/ml together with Oxa at 75 µg/ml) addition in experiment 2, pre-heated at 42°C. The tubes were immediately placed in a water bath (hour 0) and were shaken at 140 rpm at 42°C. Every time when the number of cells reached approximately 5×10^8^-10^9^/ml in earlier hours, cultures were diluted 10 fold with 5 ml of the same broth preheated to 42°C. At hour 24 (or at hour 12), aliquots of cultures were appropriately diluted and plated onto LB agar plate supplemented with appropriate antibiotics at the following concentrations: Amp at 50 µg/ml; Oxa at 150 µg/ml; Cm at 15 µg/ml; Km at 50 µg/ml. Screening and selection process performed on the plates is shown in Fig.2C. For example, single colonies that appeared on the selective plate after overnight incubation at 42°C were picked and streaked onto three individual plates containing Amp, Cm, or Km and again incubated overnight at 42°C to determine their resistance pattern. Clones resistant to two of the three drugs and sensitive to one drug were then inoculated into LB broth containing appropriate antibiotics and cultured overnight at 42°C. These cultures were used to check phage restriction, DNA isolation for southern analysis, and permanent stock preparation.

### DNA analysis

From the above experiments, the clones that showed resistance to two drugs and sensitivity to one drug were chosen from different tubes. Only one clone of the same drug-resistance pattern was chosen from each tube. Each clone was grown overnight in LB supplemented with appropriate antibiotics overnight at 42^o^C. Total DNA was isolated [39] and subjected to digestion with Bpu1101I, and separated by 0.8% agarose gel electrophoresis. After transfer to positively-charged membranes, Southern hybridization was carried out by the fluorescent ECL random prime labeling (Amersham) using BamHI-BamHI fragment containing PaeR7I RM gene derived from pSO429. Inverse PCR was performed as described [21]–[22] to amplify the two joints between the PaeR7I RM insert and the chromosome. To amplify the PaeR7I R gene side junction, genomic DNA was first cleaved with HindIII or EcoT22I upstream of the PaeR7I M gene. To amplify the PaeR7I M gene side junction, the DNA was cleaved with HincII between PaeR7I R and Amp gene and in Amp gene. After circularization of the fragments carrying a junction (Toyobo Ligation Kit), 10 ng of DNA was amplified using the LA Taq polymerase set (Takara) and 10 pico mol each of appropriate primers. All the sequences of primers used are listed in Table S3. The amplified DNA was sequenced in a sequencer (ABI), by TAKARA sequencing service or by DNA sequence service division of Human Genome Center at Institute of Medical Science, with appropriate primers.

### Reconstruction experiments

P1 transduction was performed to transfer Tet^r^ marker using BIK800 ( =  NK5992 ( =  F-, Lam^-^, IN(*rrnD-rrnE*)1 *rph-1*+ *argA81::*Tn*10*)) as a donor and R^+^ transposition product (R^+^11(BNT443) in Table S1) and R^−^ transposition product (R^−^1 (BNT390) in Table S1) as recipients to make BNT1080 (Tet^r^ version of BNT443) and BNT1081 (Tet^r^ version of BNT390). The temperature shift experiment was performed exactly in the same way as experiment series 2 until hour 12 using BNT343 ( =  MG1655/pSO429) (Tube 1) and BNT346 ( =  MG1655/pSO431) (Tube 4). All the measurements were in duplicate. Cell viability (number of colonies appeared on LB agar containing Amp (25 µg/ml)) and plasmid-carrying cells (number of colonies appeared on LB agar containing Amp (50 µg/ml), Oxa (150 µg/ml) and Cml (20 µg/ml)) were measured by incubating plates at 30°C for 20 hours. When the cultured cells approach stationary phase, ten-fold dilution were performed. At hour 12, into the 5ml aliquot of Tube 1 and Tube 4 (10^7^/ml), approximately 50/ml transductant cells (BNT1080 orBNT1081) grown to log phase were added. Tube 2: Tube 1 (R^+^) + BNT1080 (R^+^). Tube 3: Tube 1 (R^+^) + BNT1081 (R^−^). Tube 5: Tube 4 (R^−^) + BNT 1080 (R^+^). Tube 6: Tube 4 (R^−^) + BNT 1081 (R^−^). Aliquots from all 6 tubes were spread onto agar plates supplemented with Amp (50 µg/ml), Oxa and Cml, or Tet, and incubated at 42°C for 20 hours. Cell viability and number of plasmid-carrying cells were measured in the same way as described above. Aeration of the liquid cultures was continued at 42^o^C until hour 24, then, thehe aliquots of hour 24 cultures were assayed as described above, and the number of Amp and Cml resistant but Km sensitive cells at 42°C and Tet-resistant cells at 42°C were plotted.

## Supporting Information

Table S1 **Detailed information of transposition clones from experiment series 1 and 2.**
(DOC)

Table S2 **Bacterial strains and plasmids used.**
(DOC)

Table S3 **Primers used in this study.**
(DOC)

Table S4 **Effect of Mitomycin C addition.**
(DOC)

Figure S1 **Integration of the PaeR7I RM gene complex into the chromosome (Southern analysis).** Chromosomal DNA isolated from the RM transposition clones obtained from experiment series 2 (Figure 3B) was digested with Bpu1102I, electrophoresed through 0.8% agarose, transferred to membrane and probed with a BamHI-BamHI fragment of pSO429 containing PaeR7I RM gene complex (Figure 2A). There is one site near 3′ end of PaeR7I M gene, so that an integration event involving PaeR7I RM complex is expected to produce two positive bands. The difference in their mobility depends on the distance between Bpu1102I restriction site within PaeR7I M gene and two chromosomal Bpu1102I sites flanking the insertion point. Black box on the left is the original agarose gel image stained with ethidium bromide and photographed under UV to show the size markers. The Southern result in the right was obtained from the same gel. The first number indicates the culture tube, while the second the clone. The clones 2-1, 2-2 and 8-1, 8-2 were obtained from the same tube but showed a different antibiotic-resistance pattern. Two vertical lines in the middle indicate omission of several lanes of sister clones with an identical pattern from one tube.
(TIFF)

Figure S2 **Sequences of the transposition products (i).**Left - Apparent co-integrate with IS1 (at co-ordinate 19796-20563) type. Right - Apparent co-integrate with IS1 (at co-ordinate 1977258-1977294) type. Transposition regions where the plasmid (all or partially) is integrated into chromosome are sequenced and shown. The clone numbers correspond to those shown in Table S1. Blue, plasmid; Black, chromosome; TD, target duplication; Asterisk, identical base pairs; IRR, inverted repeat at the right end of IS1 (IS5); IRL, inverted repeat at the left end of IS1 (IS5); Green arrow, chromosomal genes; Green transparent squares, duplicated regions.
(TIFF)

Figure S3 **Sequences of the transposition products (ii).** Left - Apparent co-integrates with IS5 type. Right - Apparent co-integrate type with IS1 and IS5 from R- and R+ version obtained from experiment series 2. Transposition regions where the plasmid (all or partially) is integrated into chromosome are sequenced and shown. The clone numbers correspond to those shown in Table S1. Blue, plasmid; Black, chromosome; TD, target duplication; Asterisk, identical base pairs; IRR, inverted repeat at the right end of IS1 (IS5); IRL, inverted repeat at the left end of IS1 (IS5); Green arrow, chromosomal genes; Green transparent squares, duplicated regions.
(TIFF)

Figure S4 **Sequences of the transposition products (iii).** Left and Right - Denovo insertion. Transposition regions where the plasmid (all or partially) is integrated into chromosome are sequenced and shown. The clone numbers correspond to those shown in Table S1. Blue, plasmid; Black, chromosome; TD, target duplication; Asterisk, identical base pairs; IRR, inverted repeat at the right end of IS1(IS5); IRL, inverted repeat at the left end of IS1 (IS5); Green arrow, chromosomal genes; Green transparent squares, duplicated regions.
(TIFF)

Figure S5 **Sequences of the transposition products (iv).** Left and Right - Crossing-over with short homology Transposition regions where the plasmid (all or partially) is integrated into chromosome are sequenced and shown. The clone numbers correspond to those shown in Table S1. Blue, plasmid; Black, chromosome; TD, target duplication; Asterisk, identical base pairs; IRR, inverted repeat at the right end of IS1(IS5); IRL, inverted repeat at the left end of IS1 (IS5); Green arrow, chromosomal genes; Green transparent squares, duplicated regions.
(TIFF)

Figure S6 **Pulsed-field gel electrophoresis.** Cell cultures at O.D.0.6 each of MG1655(no plasmid), MG1655(with pSO429 (R+)), and MG1655(with pSO431(R^−^)) after 0, 6, 24 hours of heat induction were subjected to pulsed-field gel electrophoresis in a CHEF-DR III System (Bio-Rad). Each plug was prepared in 1% agarose and placed into each well of 1.2% Certified Megabase agarose (Bio-Rad) and run in Tris-borate-EDTA buffer (0.045 M Tris-borate, 0.01 M EDTA) under following condition: 18 hour run time, 5- to 40-s of switch time ramp, 120° included angle, 6 V/cm, at 14°C. For size markers, 50 kb DNA ladder (Bio-Rad) and λ DNA/HindIII markers were used. After the run, the gel was stained with ethidium bromide for 1 h, destained in water, and the fluorescence response was examined using a FLA-5100 image analyzer (Fujifilm, Minato-ku, Tokyo, Japan).
(TIFF)
