# Supplementary material for: IS-Linked Movement of a Restriction-Modification System
Source: PLoS One. 2011 Jan 31;6(1):e16554. doi: 10.1371/journal.pone.0016554 (PMC3031569; doi:10.1371/journal.pone.0016554)
Supplement: Table S1 — Detailed information of transposition clones from experiment series 1 and 2. (DOC) [file pone.0016554.s001.doc]

| Type | Numbers found | Experiment | PaeR7I  R+/- M+  on starting  plasmid | Insertion  position of the recipient | Numbers found | (direction of the donor insertion*1):  donor sequence (Target duplication  sequences are capitalized) : (Gene) | Transposition product name |
| --- | --- | --- | --- | --- | --- | --- | --- |
| (1).Apparent cointegrate types with IS1 | 19 | 1 | R+ M+ | IS1 at 19796-  20294 | 10 | (+)：5’ gttACCAATGCTtaa：(in Amp) | EI |
|  |  |  |  |  |  | (+)：5’ attGAAGCATTTatc：(in Amp) | EJ |
|  |  |  |  |  |  | (+)：5’gcaTAATTCTCtac：(in Amp) | EN |
|  |  |  |  |  |  | (+)：5’aaaGGCCGGATAAaac：(in Cm) | E2 |
|  |  |  |  |  |  | (+)：5’catCGAGCATCAaat：(in Km) | E11 |
|  |  |  |  |  |  | (-)：5’tatGTATCCGCCTatg：(in Amp) | E12 |
|  |  |  |  |  |  | (+)：5’atcAGGATTATCaat：(in Km) | E15 |
|  |  |  |  |  |  | (+)：5’gaaATCGTCGTGGgta：(in Cm) | E23 |
|  |  | 2 | R+ M+ |  |  | (-) : 5'ataCAatc : (in Km ) | R+12 |
|  |  |  | R- M+ |  |  | (+) : 5'aagAAGTTGTCcat : (in Cm) | R-9 |
|  |  | 1 | R+ M+ | IS1 at 1976527-  1977294 | 9 | (+)：5’gttGTAATTCATtaa：(in Cm) | EA |
|  |  |  |  |  |  | (-)：5’taaCTGGCGAAtac：(in Amp) | EK |
|  |  |  |  |  |  | (-)：5’attTGATGCTCGatg：(in Km) | E1 |
|  |  |  |  |  |  | (+)：5’cggAAATGTTaat：(in Amp) | E6 |
|  |  |  |  |  |  | (-)：5’ctgGTGAAAGTaaa：(in Amp) | E16 |
|  |  |  |  |  |  | (-)：5’gccTTCCTGTTttt：(in Amp) | E25 |
|  |  | 2 | R+ M+ |  |  | (+) : 5'aacAGGAATCGaat : (in Km) | R+11 |
|  |  |  | R- M+ |  |  | (-) : 5'attCGATTCCTgtt : (in Km) | R-1 |
|  |  |  | R- M+ |  |  | (+) : 5'tatCACCAGCTCAacc : (in Cm) | R-6 |
| (2).Apparent cointegrate types with IS5 | 5 | 2 | R- M+ | IS5 at 27379-  274373 | 2 | (+) : 5'attcttCTAAtacctg: (in Km) | R-3 |
|  |  |  |  |  |  | (+) : 5'attcttCTAAtacctg: (in Km) | R-4 |
|  |  | 1 | R+ M+ | IS5 at 687074-  688268 |  | (-)：5’aaccctTTAGggaaat：(in Cm) | EH |
|  |  |  | R+ M+ | IS5 at 3363191-  3364385 | 1 | (-)：5’aattctCTTActgtca：(in Amp) | E10 |
|  |  | 2 | R- M+ | IS5 at 273179-  274373 | 1 | (+) : 5'attcttCTAAtacctg: (in Km) | R-7 |
| (3).aberrant insertion with IS | 2 | 1 | R+ M+ | 2677075 | 1 |  | E20 |
|  |  |  | R+ M+ | 4249594 | 1 |  | E3 |
| (4).Crossing- over using very short homology | 2 | 1 | R+ M+ | 1864676 | 1 |  | E5 |
|  |  |  | R+ M+ | 2719998 | 1 |  | EB |
| *1　Sequences are shown clock-wise direction along with E.coli DNA sequence. | | | | | | | |

Table S1. Detailed information of transposition clones from experiment series 1 and 2
