# Supplementary material for: IS-Linked Movement of a Restriction-Modification System
Source: PLoS One. 2011 Jan 31;6(1):e16554. doi: 10.1371/journal.pone.0016554 (PMC3031569; doi:10.1371/journal.pone.0016554)
Supplement: Table S2 — Bacterial strains and plasmids used. (DOC) [file pone.0016554.s002.doc]

Table S2. *E. coli* strains and plasmids used

| Strain or plasmid | | Relevant properties | Comments | Source /reference |
| --- | --- | --- | --- | --- |
| Strains | MG1655 | *F-, lambda-,rph-1* | K12 strain sequenced | F.R.Blattner [29] gift from D.Biek |
|  | NK5992 | *F-, lambda-, argA81::Tn10, IN(rrnD-rrnE)1* | Tet^r^ | N. Kleckner |
|  | BNT443 | As MG1655 but with IS1-mediated insertion of pSO429 | PaeR7I R+M+, Amp^r^, Cm^r^ | This study |
|  | BNT390 | As MG1655 but with IS1-mediated insertion of pSO431 | PaeR7I R-M+, Amp^r^, Cm^r^ | This study |
|  | BNT1080 | As BNT443 but *argA81::Tn10* | P1 from NK5992. Tet^r^ | This study |
|  | BNT1081 | As BNT390 but *argA81::Tn10* | P1 from NK5992. Tet^r^ | This study |
|  | MDS42 | As MG1655 but all IS elements along with 704 genes deleted |  | Posfai et al [40]  Purchased from Scarab Genomics |
| Plasmids | pHSG415 | pSC101ts replicon, Amp^r^ Km^r^ Cm^r^, cloning vector (7.1 kb) |  | Hashimoto-Gotoh (1981) [28] |
|  | pTN5 | pBR322 PaeR7I R+ M+ Amp^r^ | Insertion of a BamHI linker into pPAORM3.8 [3] [27] cut with NruI | T.Naito |
|  | pSO421 | PSC101ts replicon, Amp^r^ Km^r^ Cm^r^, del. IS1 region (141bp) |  | This study |
|  | pSO429 | As pSO421, but PaeR7I R+ M+ |  | This study |
|  | pSO431 | As pSO429, but PaeR7I R- M+ |  | This study |
