# Supplementary material for: IS-Linked Movement of a Restriction-Modification System
Source: PLoS One. 2011 Jan 31;6(1):e16554. doi: 10.1371/journal.pone.0016554 (PMC3031569; doi:10.1371/journal.pone.0016554)
Supplement: Table S3 — Primers used in this study. (DOC) [file pone.0016554.s003.doc]

Table S3. Primers used in this study

| IPCR primers | 429R7r-1, 5'cggcacctacttgatgaatg |
| --- | --- |
|  | 429R7m-1, 5'cccaaatcatcgacctcagt |
|  | 421seq13, 5'ttcgaaagcaaattcgacccgg |
|  | R7m-3, 5'cccatcatccagccagaaagtg |
|  | IPCR23R7-A, 5'cacgcggtatgacttgctgtgtc |
|  | 421seq18, 5'ggcagtgaatgggggtaaatggca |
|  | 421seq22, 5'aagggcaccaataactgcctta |
|  |  |
| Sequence primers | 429R7m-3, 5'catcatccagccagaaagtg |
|  | 421seq-15, 5'cgttttccaatgatgagcac |
|  | 421seq-16, 5'caacggaaattgctcatcag |
|  | IPCR-Ap-C, 5'gatggaggcggataaagttg |
|  | 421seq-28, 5'ggagaagctcaacgaactgg |
|  | IPCR-Ap-E, 5'gtggtcctgcaactttatcc |
|  | IPCR-Ap-B 5'cgatcaaggcgagttaca |
|  | 421seq-13, 5'ttcgaaagcaaattcgacc |
|  | IPCR20-R7-C, 5'gtccttttaacagcgatcgc |
|  | 429R7m-4, 5'ggatcgcagtggtgagtaac |
|  | 421seq-12, 5'ggcaatgaaagacggtgag |
|  | 421seq-18, 5'cagtgaatgggggtaaatgg |
|  | 421seq-5, 5'cgtttcagtttgctcatgg |
|  | 421seq-22, 5'gggcaccaataactgcctt |
|  |  |
|  | KsCHII-1, 5'caagtcagcgtaatgc |
|  | KsHdIII-1, 5'caataaaactgtctgcttac |
|  | CsCHII-1, 5'gccccgccctgccactcatc |
|  | CsHdIII-1, 5'ctaaggaagctaaaatggag |
|  | InsAout, 5'gcgggctgttgcccggcatc |
|  | InsBout, 5'cacctggcacggctgggacg |
|  | Trs5R, 5'ccaactggcgatgttattcac |
|  | Trs5L, 5'gcgaaggtaagttgatgactc |
