# Supplementary material for: IS-Linked Movement of a Restriction-Modification System
Source: PLoS One. 2011 Jan 31;6(1):e16554. doi: 10.1371/journal.pone.0016554 (PMC3031569; doi:10.1371/journal.pone.0016554)
Supplement: Table S4 — Effect of Mitomycin C addition. (DOC) [file pone.0016554.s004.doc]

Table S4. Effect of mitomycin C on transposition

| Transposition efficiency with Mitomycin C addition | | | |
| --- | --- | --- | --- |
|  | | Mitomycin C (10 ng/ml) | |
|  | Experiment | - | + |
| R^+^ | 1 | 1.56.E04 | 5.3.E03 |
|  | 2 | 1.91E04 | 2.7.E03 |
| R^-^ | 1 | 3.4.E07 | 1.21.E04 |
|  | 2 | 4.3.E07 | 3.9.E04 |

Temperature-shift experiment was carried out at 42°C in the presence of Amp (at 25μg/ml together with Oxa at 75μg/ml) as described. At seven hour after the shift, mitomycin C was added 10ng/ml to both R^+^ and R^−^ culture and aerated for 1 hour. The culture was then washed to remove mitomycin C, resuspended with LB containing Amp at 42°C and aerated until hour 24. The transposition frequency was measured at hour 24.
