# Supplementary figures and images for: IS-Linked Movement of a Restriction-Modification System
Source: PLoS One. 2011 Jan 31;6(1):e16554. doi: 10.1371/journal.pone.0016554 (PMC3031569; doi:10.1371/journal.pone.0016554)

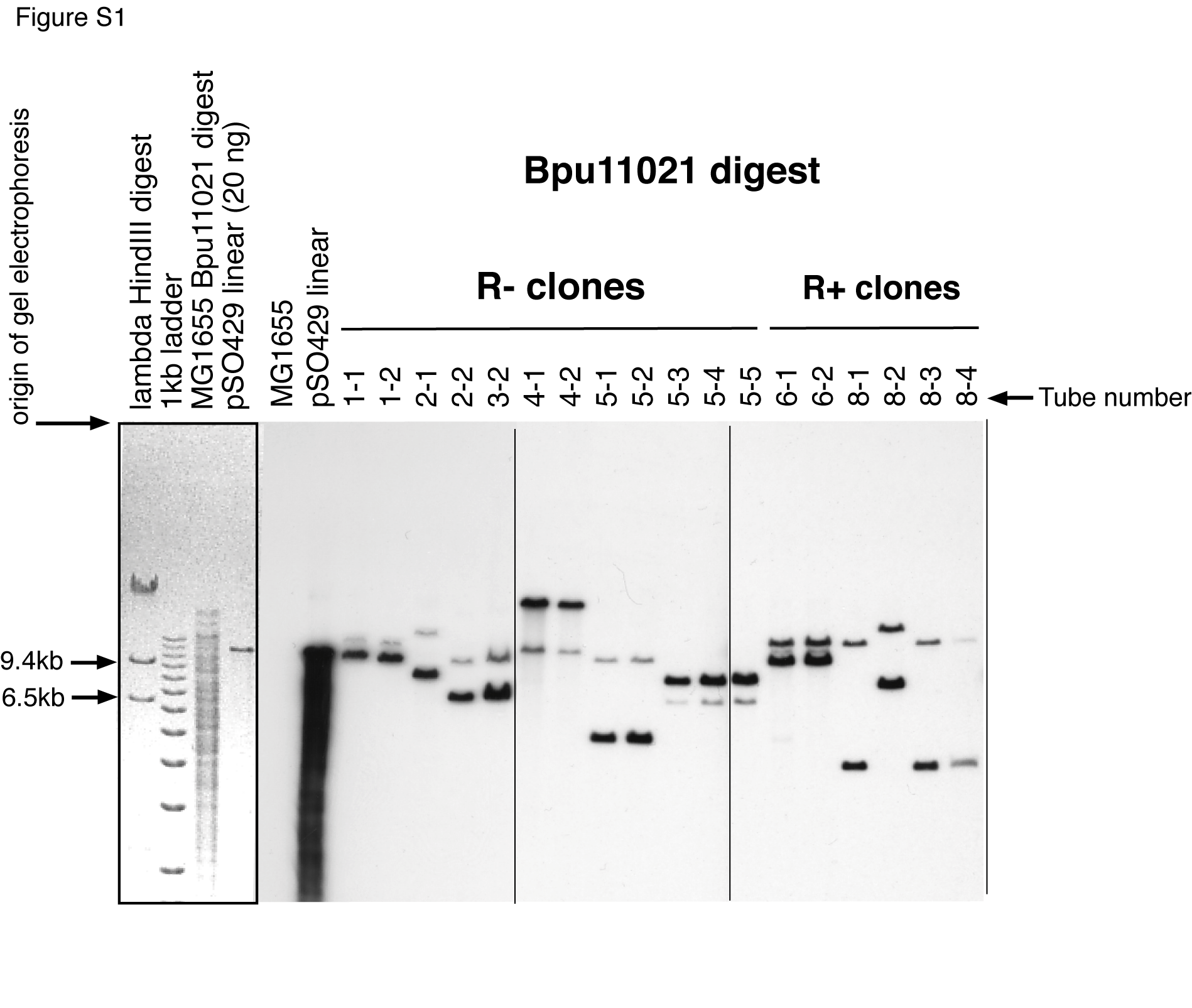

Supplement: Figure S1 — Integration of the PaeR7I RM gene complex into the chromosome (Southern analysis). Chromosomal DNA isolated from the RM transposition clones obtained from experiment series 2 (Figure 3B) was digested with Bpu1102I, electrophoresed through 0.8% agarose, transferred to membrane and probed with a BamHI-BamHI fragment of pSO429 containing PaeR7I RM gene complex (Figure 2A). There is one site near 3′ end of PaeR7I M gene, so that an integration event involving PaeR7I RM complex is expected to produce two positive bands. The difference in their mobility depends on the distance between Bpu1102I restriction site within PaeR7I M gene and two chromosomal Bpu1102I sites flanking the insertion point. Black box on the left is the original agarose gel image stained with ethidium bromide and photographed under UV to show the size markers. The Southern result in the right was obtained from the same gel. The first number indicates the culture tube, while the second the clone. The clones 2-1, 2-2 and 8-1, 8-2 were obtained from the same tube but showed a different antibiotic-resistance pattern. Two vertical lines in the middle indicate omission of several lanes of sister clones with an identical pattern from one tube. (TIFF) [file pone.0016554.s005.tif]

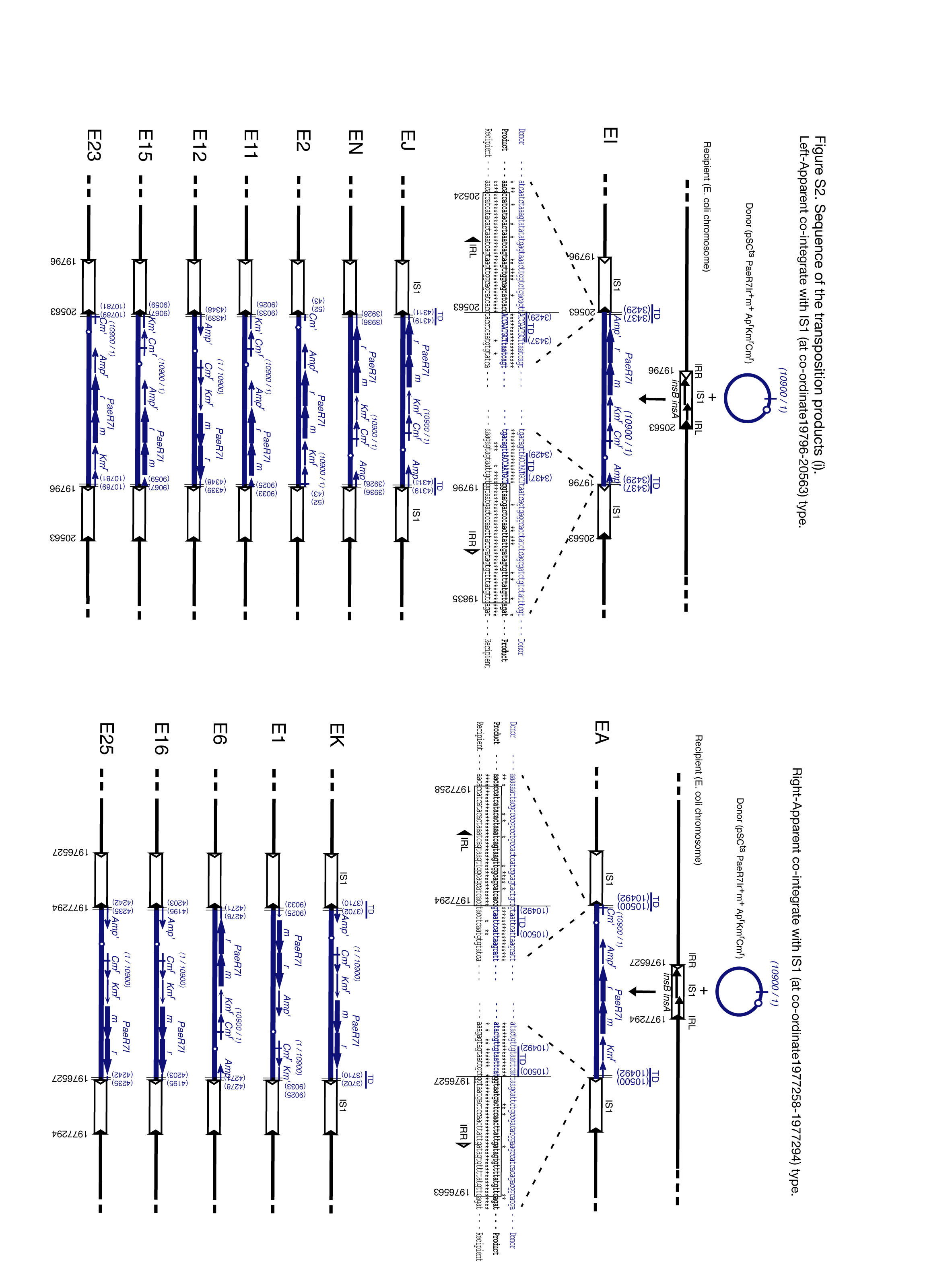

Supplement: Figure S2 — Sequences of the transposition products (i).Left - Apparent co-integrate with IS1 (at co-ordinate 19796-20563) type. Right - Apparent co-integrate with IS1 (at co-ordinate 1977258-1977294) type. Transposition regions where the plasmid (all or partially) is integrated into chromosome are sequenced and shown. The clone numbers correspond to those shown in Table S1. Blue, plasmid; Black, chromosome; TD, target duplication; Asterisk, identical base pairs; IRR, inverted repeat at the right end of IS1 (IS5); IRL, inverted repeat at the left end of IS1 (IS5); Green arrow, chromosomal genes; Green transparent squares, duplicated regions. (TIFF) [file pone.0016554.s006.tif]

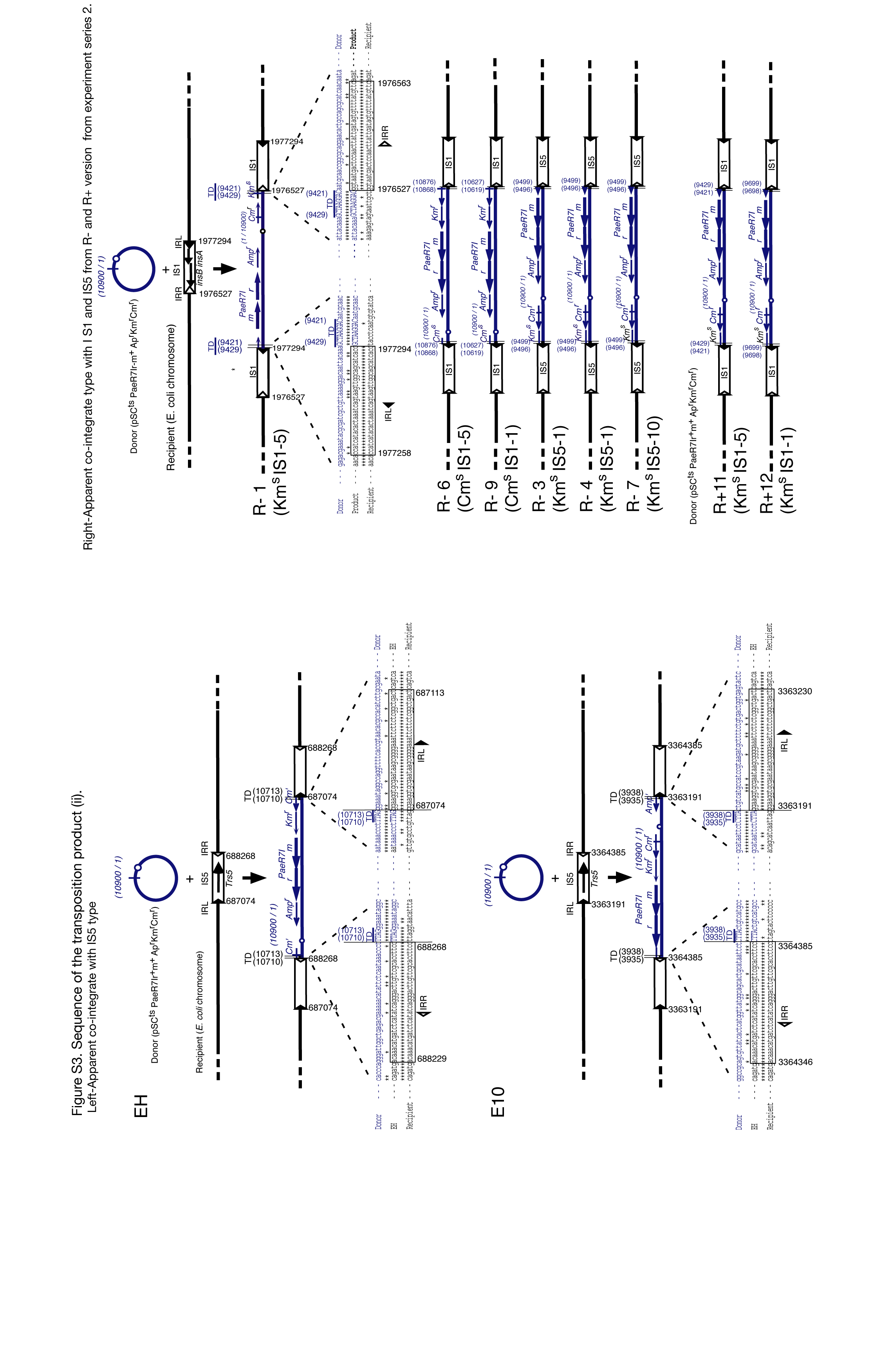

Supplement: Figure S3 — Sequences of the transposition products (ii). Left - Apparent co-integrates with IS5 type. Right - Apparent co-integrate type with IS1 and IS5 from R- and R+ version obtained from experiment series 2. Transposition regions where the plasmid (all or partially) is integrated into chromosome are sequenced and shown. The clone numbers correspond to those shown in Table S1. Blue, plasmid; Black, chromosome; TD, target duplication; Asterisk, identical base pairs; IRR, inverted repeat at the right end of IS1 (IS5); IRL, inverted repeat at the left end of IS1 (IS5); Green arrow, chromosomal genes; Green transparent squares, duplicated regions. (TIFF) [file pone.0016554.s007.tif]

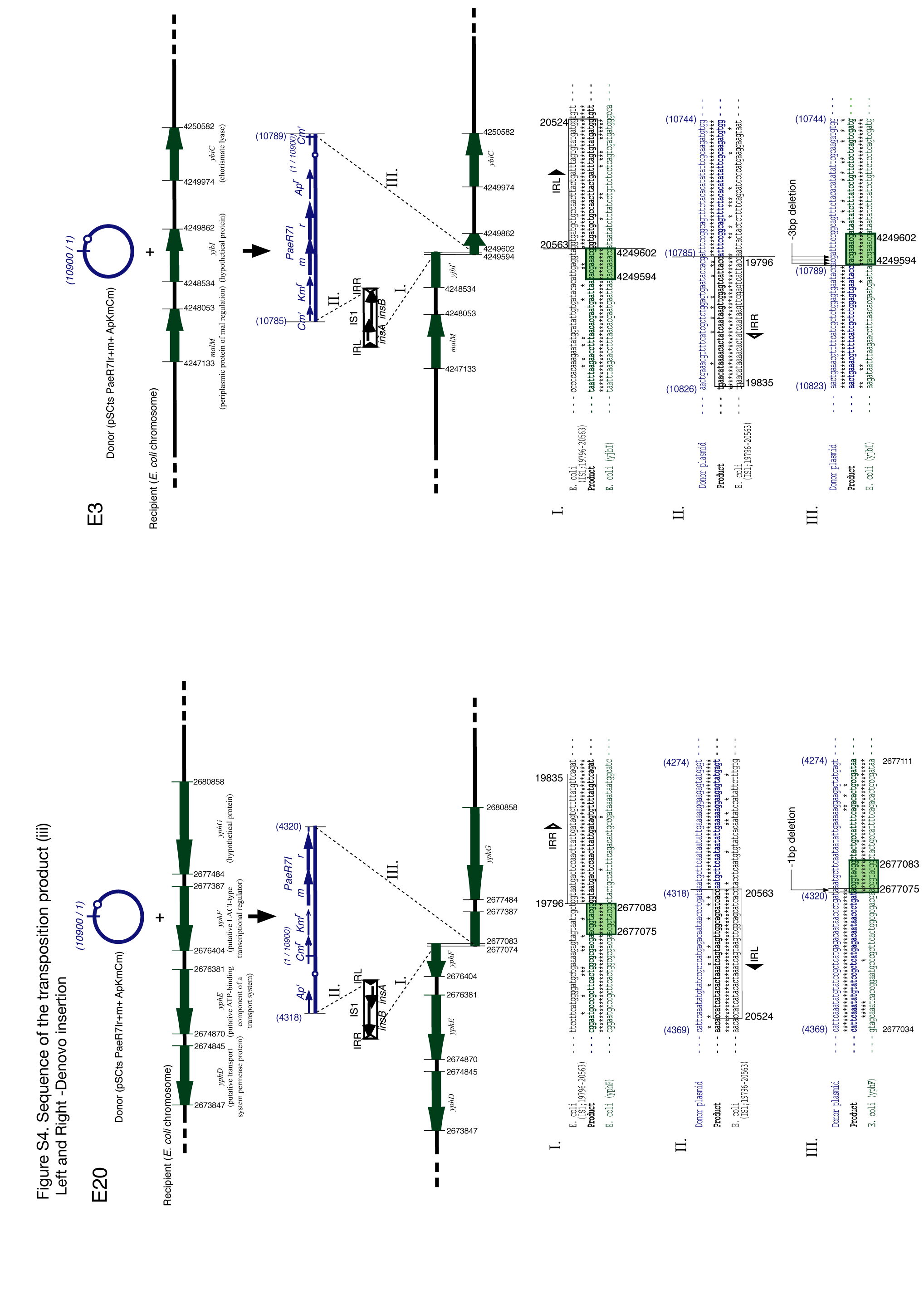

Supplement: Figure S4 — Sequences of the transposition products (iii). Left and Right - Denovo insertion. Transposition regions where the plasmid (all or partially) is integrated into chromosome are sequenced and shown. The clone numbers correspond to those shown in Table S1. Blue, plasmid; Black, chromosome; TD, target duplication; Asterisk, identical base pairs; IRR, inverted repeat at the right end of IS1(IS5); IRL, inverted repeat at the left end of IS1 (IS5); Green arrow, chromosomal genes; Green transparent squares, duplicated regions. (TIFF) [file pone.0016554.s008.tif]

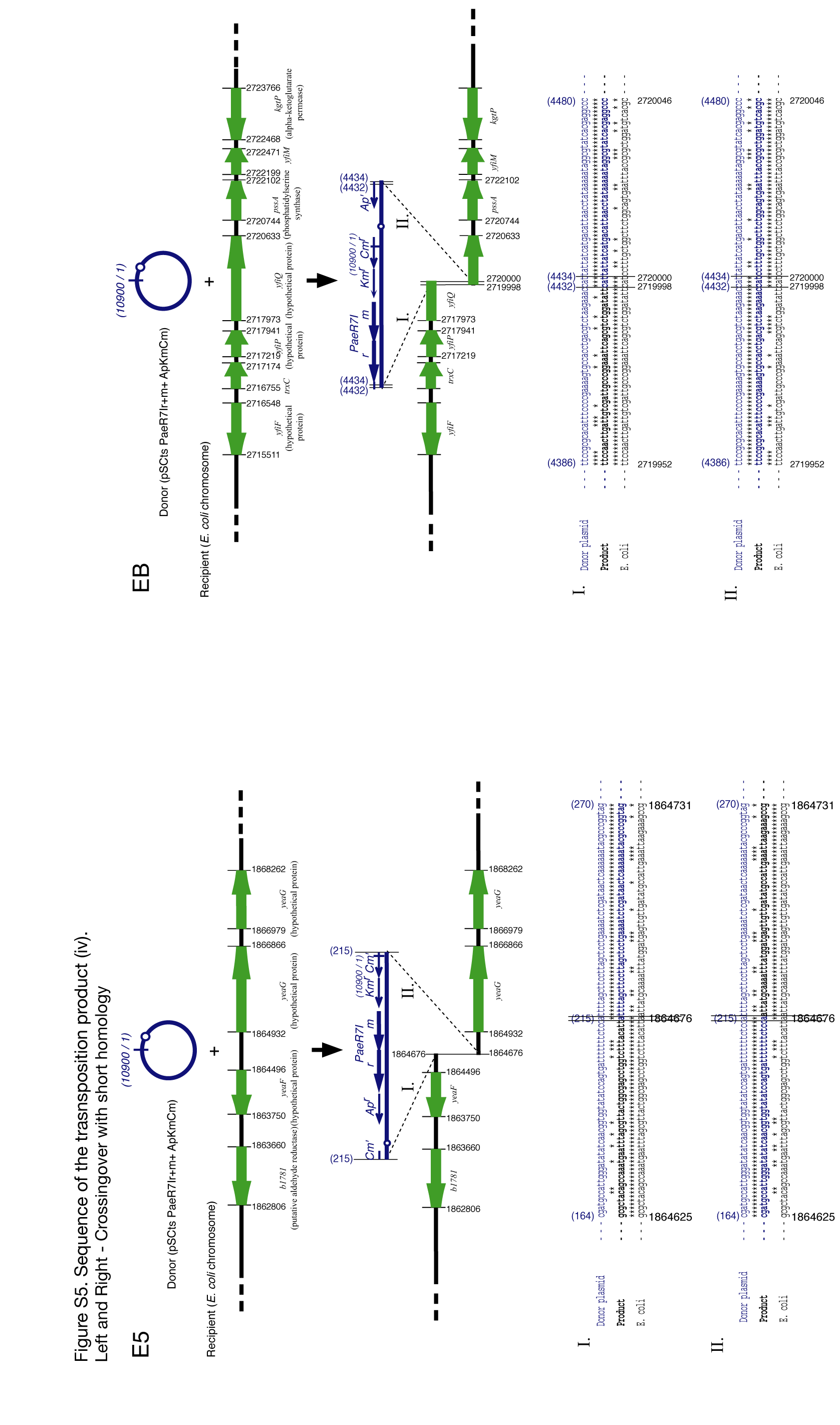

Supplement: Figure S5 — Sequences of the transposition products (iv). Left and Right - Crossing-over with short homology Transposition regions where the plasmid (all or partially) is integrated into chromosome are sequenced and shown. The clone numbers correspond to those shown in Table S1. Blue, plasmid; Black, chromosome; TD, target duplication; Asterisk, identical base pairs; IRR, inverted repeat at the right end of IS1(IS5); IRL, inverted repeat at the left end of IS1 (IS5); Green arrow, chromosomal genes; Green transparent squares, duplicated regions. (TIFF) [file pone.0016554.s009.tif]

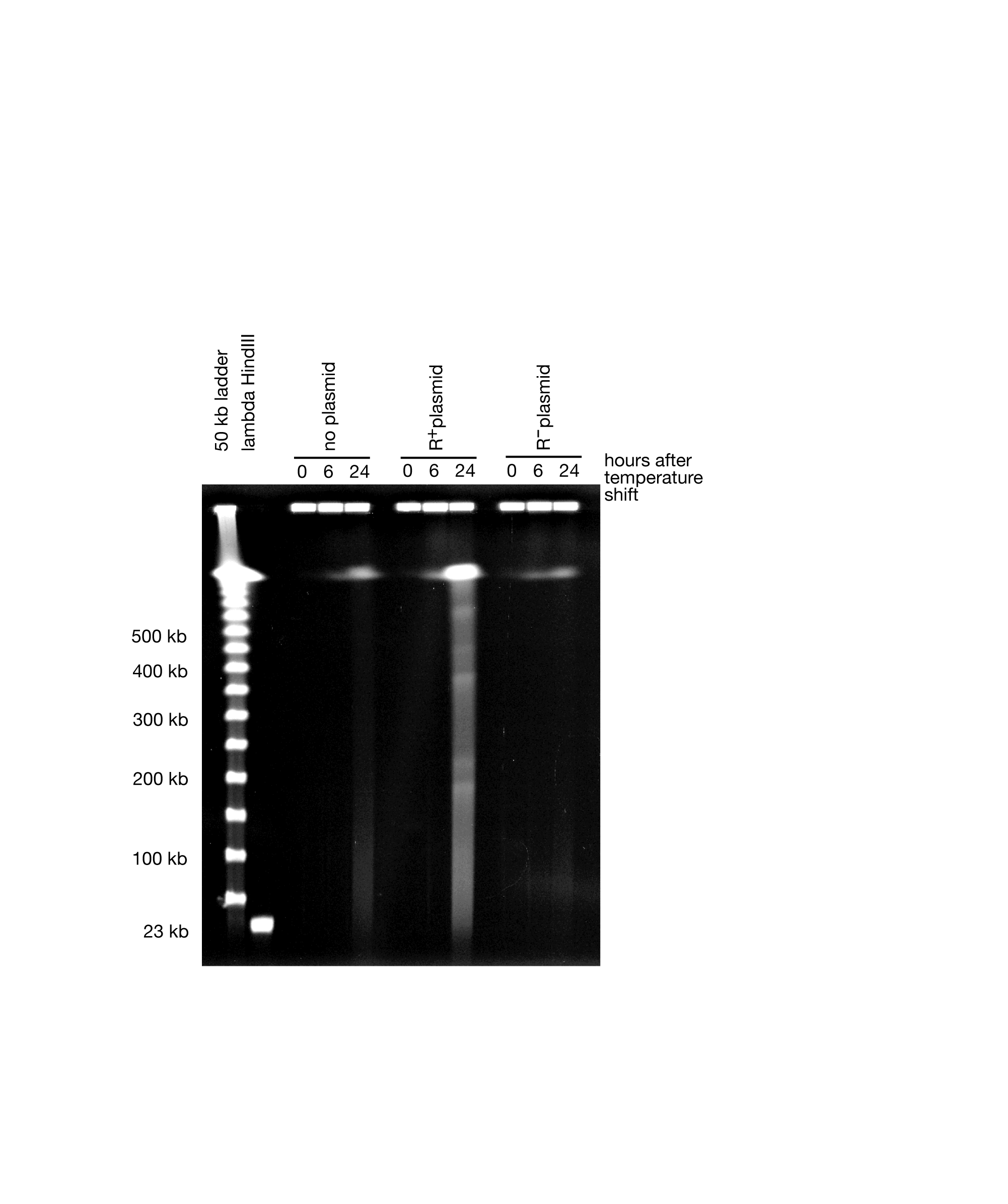

Supplement: Figure S6 — Pulsed-field gel electrophoresis. Cell cultures at O.D.0.6 each of MG1655(no plasmid), MG1655(with pSO429 (R+)), and MG1655(with pSO431(R−)) after 0, 6, 24 hours of heat induction were subjected to pulsed-field gel electrophoresis in a CHEF-DR III System (Bio-Rad). Each plug was prepared in 1% agarose and placed into each well of 1.2% Certified Megabase agarose (Bio-Rad) and run in Tris-borate-EDTA buffer (0.045 M Tris-borate, 0.01 M EDTA) under following condition: 18 hour run time, 5- to 40-s of switch time ramp, 120° included angle, 6 V/cm, at 14°C. For size markers, 50 kb DNA ladder (Bio-Rad) and λ DNA/HindIII markers were used. After the run, the gel was stained with ethidium bromide for 1 h, destained in water, and the fluorescence response was examined using a FLA-5100 image analyzer (Fujifilm, Minato-ku, Tokyo, Japan). (TIFF) [file pone.0016554.s010.tif]
